# Exploring the health information seeking behavior of social media users under the background of COVID-19 pandemic: An empirical study based on social cognitive theory

**DOI:** 10.3389/fpsyg.2022.1045914

**Published:** 2022-11-08

**Authors:** Xiaoyi Zhang, Beibei Chen, Guowang Li, Yueqi Dong

**Affiliations:** ^1^School of Biological and Agricultural Engineering, Jilin University, Changchun, China; ^2^School of Business, Dalian University of Technology, Dalian, China

**Keywords:** COVID-19, social media users, health information seeking behavior, social cognitive theory, emotional arousal, self-efficacy

## Abstract

With the outbreak of COVID-19 in late 2019, people’s awareness of actively searching for health information has been growing. Coupled with the promotion of “Internet + medical and health,” social media, as an important platform for health information dissemination, has become one of the important information sources for users to obtain health information. However, health information seeking behavior in public health emergencies are quite different from those in daily life. Therefore, the research question of this paper is to explore the influencing factors of health information seeking behavior of social media users in the context of COVID-19. To this end, based on the research framework of social cognition theory, this paper selects six variables to construct a structural equation modeling, including information and platform quality, experience, social support, emotional arousal, self-efficacy, and social media users’ health information seeking behavior. The empirical study is carried out by collecting 219 valid questionnaires. The research results show that: (1) Information and platform quality, and use experience have a significant positive effect on emotional arousal; (2) Experience, social support, and emotional arousal have a significant positive effect on self-efficacy; (3) Emotional arousal and self-efficacy have a significant positive effect on social media users’ health information seeking behavior. The research provides an important theoretical reference for social media users and operators to cope with the huge demand for health information in the post-COVID-19 era.

## Introduction

COVID-19 is a disease caused by severe acute respiratory syndrome coronavirus 2 (SARS-CoV-2). The outbreak of COVID-19 in late 2019 has posed a severe challenge to global public health (Liu et al., 2021). During public health emergencies, in order to alleviate panic, people generally use online information, social media and other online ways to search for health information to alleviate negative emotions (Tausczik et al., 2012). Long-term coexistence with coronavirus is an important feature of the post-epidemic era. During the COVID-19 pandemic, timely access to health care information and necessary preventive measures can reduce public anxiety and help minimize negative mental impacts (Zhao et al., 2020). In the context, the importance of citizens’ attention to health information and the importance of guiding citizens to efficiently seeking for health information have reached an unprecedented height. The COVID-19 pandemic has boosted the development of online health care, increasing the need for people to access information from the Internet (Li and Liu, 2020).

The outbreak of the new crown epidemic has led to an increasing awareness of users actively seek health information. At the same time, the development of network technology also provides an important platform support for it. With the advancement of “Internet + medical and healthcare,” various health service platforms continue to develop. As an important platform for health information dissemination, social media has become one of the important information sources for users to obtain health information (Li and Liu, 2020). At the same time, due to the diverse types of social media, which have the characteristics of immediacy, diversity, and autonomy (Hwang and Kim, 2015), information users of different occupations and different age groups can inquire and use the required health information from it. Seeking for health information through social media has gradually become an important supplement to traditional information seeking. The Internet is the fastest developing carrier of health information and the largest medical library in the world. More and more governments, medical departments, enterprises and other institutions have established health information portals, and the health information based on the network is becoming more and more abundant (Morahan-Martin, 2004; Li et al., 2015). According to relevant data from Baidu Index from January to September 2022, the average daily search index for keywords “health” and “medical” is 5,186 (5,000+ is the hot search keyword). Therefore, under the background of the epidemic and the development trend of “Internet + medical and healthcare,” users will actively seek for health information through social media more frequently. However, in this process, the user’s own cognitive type, personal information literacy, information quality and the retrieval tools used will lead to large differences in the process and results of information seeking behavior. Therefore, how to explore and reveal the influencing factors and correlations of their health information seeking behaviors for a typical group of social media users has increasingly become the focus of academic attention.

The health information seeking behavior during public health emergencies is very different from that in daily life. Firstly, the environmental factors affecting the information seeking behavior of public health emergencies are often more complex than those in conventional scenarios (Hsu, 2021). Secondly, the COVID-19 pandemic has seen a huge increase in public demand for health information (Zhao et al., 2020). Finally, the richness of health information and transmission patterns during this pandemic has also changed (Hsu, 2021). However, the existing research lacks relevant empirical evidence on the health information seeking behavior of social media users caused by public health emergencies. Therefore, the research question of this paper is to explore the influencing factors of health information seeking behavior of social media users in the context of the COVID-19. Based on this, in order to help user, obtain health information efficiently and promote the construction of a healthy country, this paper takes social media users of different backgrounds as the main research objects, adopts quantitative research methods, and establishes a structural equation model of social media users’ health information seeking behavior. This paper uses this model to explore the health information seeking behavior of social media users, and provide theoretical support for social media users to effectively obtain health information, provide reference for hospitals, health information professionals and relevant government departments, so as to provide important empirical evidence for responding to similar public health emergencies in the future.

## Literature review

Health information generally refers to all knowledge, techniques, concepts and behaviors related to people’s health care and balanced nutrition before illness, medical recovery and patient services during illness in terms of physiology or psychology (Lin and Chang, 2018). Information seeking can be unintentional, passive or active, and often purposeful, with individuals seeking information to satisfy personal needs or goals. Information seeking behavior refers to the behavior of searching and using information in any way according to individual needs (Hsu, 2021). Specifically, it is related to the behavior resulting from the interaction between the information source and the information demander. Health information seeking behavior refers to the behavioral process that users actively obtain health information through various information media in order to meet the health needs of specific events or situations (Xiao et al., 2014; Li and Wang, 2018). In recent years, scholars in various fields at home and abroad have achieved some results in the research on health information seeking behavior. The research on health information seeking behavior has gradually developed into a multi-disciplinary and cross-disciplinary research topic, covering information science, medicine, communication, sociology, management and other disciplines (Mirzaei et al., 2021), usually focusing on two aspects of influencing factors and behavior patterns.

### Studies on the influencing factors of health information seeking behavior

The influencing factors of users’ health information seeking behavior is one of the main issues studied by scholars. With the expansion of user groups and the extension of research environments and contexts, information behaviors and types have become increasingly rich and diversified, and interdisciplinary reference and integration have become the mainstream trend of research in this direction. Among them, systematic analysis methods such as information ecology theory, social cognition theory, and meta-analysis are used to discuss the influencing factors of health information seeking behavior, which has become one of the important research paradigms of scholars at home and abroad (Li et al., 2015).

From the perspective of information ecology, the research on the influencing factors of users’ health information seeking behavior has experienced the development process from analyzing the influence of fragmented information factors (such as information people (user group characteristics), information (information type), information technology (communication media), etc.) to analyzing the influencing factors and interaction relationships of users’ health information seeking behavior from a holistic perspective. Cao et al. (2016) analyzed the effects of perceived availability, self-efficacy, professional knowledge and ability, and reward on online health information seeking: the characteristics of information sources affect the activation mechanism, and the activation mechanism affects online health information seeking behavior. Almoajel and Almarqabi (2016) demonstrated the relationship between users’ access to timely and effective information and online health information seeking. Jiang et al. (2021) showed that perceived susceptibility, perceived severity and self-efficacy were significantly and positively correlated with the diversity of Internet health information seeking behaviors, and technological enablers were positively correlated with Internet health information seeking behaviors.

### Studies on patterns of health information seeking behavior

Studies have shown that certain group characteristics of users (such as age, education, health status, etc.) will have a significant impact on health information seeking behavior under different media (Maon et al., 2017). Therefore, scholars often take a specific user group as the research object and conduct research on the characteristics and patterns of their health information seeking behavior. Maon et al. (2017) found that the online health information seeking model consists of four parts: the behavior pattern before seeking, the initial behavior pattern, the behavior pattern during seeking and the behavior pattern after seeking. Although the health information seeking behavior of different user groups has certain differences in seeking content, information needs, and group characteristics. But their behavior patterns can be summed up as proactive, motivational and passive (Jia et al., 2021). Among them, proactive users usually have high health literacy, pay attention to health care, and some users will pay attention to health forums, websites, etc. for a long time. The health information seeking behavior of such users is “habitual.” motivational users usually have clear medical and health information needs, and need to obtain health information through different media to answer their own health problems. The health information seeking behavior of such users is “motivation-driven (for example, seeking for how to treat their own cold).” Passive users generally have no direct demand for health information, and usually encounter and discover health knowledge that interests them in daily life. At the same time, the different health information seeking behavior patterns of users are not completely independent, and most users will adopt parallel strategies, but the emphasis on different patterns may vary from person to person.

The existing literature has studied the characteristics, patterns and influencing factors of health information seeking behaviors of different user groups, but has paid little attention to the individual differences and behavioral differences of health information seeking behaviors of users on social media platforms. As the main carrier of health information and interpersonal communication, the impact of information dissemination media on users’ health information seeking has been ignored, and the existing research findings are rarely applied to the COVID-19 outbreak. Therefore, this paper takes the epidemic as the research background, takes social media users as the research object, and based on the social cognitive theory, establishes the structural equation model of the influencing factors of social media users’ health information seeking behavior. This paper innovatively considers the emotional factors of users, quantitatively analyzes the behavioral characteristics and main motivations of this group, and provides theoretical guidance for service providers to better optimize health information services based on the behavioral characteristics of social media users.

## Theoretical models and research assumptions

### Model construction based on social cognitive theory

**Figure 1:**
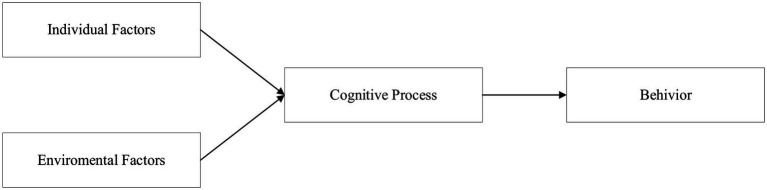
Research framework of social cognition theory.

Social cognitive theory is the basic theory about individual behavior formed by Bandura (1977) by adding cognitive part to the traditional behaviorist personality theory. The ternary interactive determinism in social cognitive theory focuses on the dynamic mutual determinism between the environment, people and their behaviors. The environmental factors, behaviors, and individual human factors are regarded as theoretical entities that are independent and interact with each other and thus determine each other. From the psychological point of view, human’s initiative is greatly influenced by subjective factors, but also by environmental factors, which is not the one-way influence of behavior and the environment. As a mature theory, social cognition theory has been widely used to explain and predict individuals and group behavior characteristics, and to seek ways to change individual or group behavior (Myrick, 2017). As a kind of human behavior, the health information seeking behavior of social media users conforms to the judgment that individual cognition and environmental effects have an impact on human behavior in theory. There is an influencing relationship path among social environmental factors, individual factors and individual action choices, and the triadic interaction model can be well applied to this study (Wang et al., 2021). Therefore, based on the ternary interactive determinism, combined with the viewpoint of information ecology, this paper focuses on the positive impact path from environmental and individual factors to cognitive processes to behavior, and establishes a research framework as shown in Figure 1.

**Figure 2:**
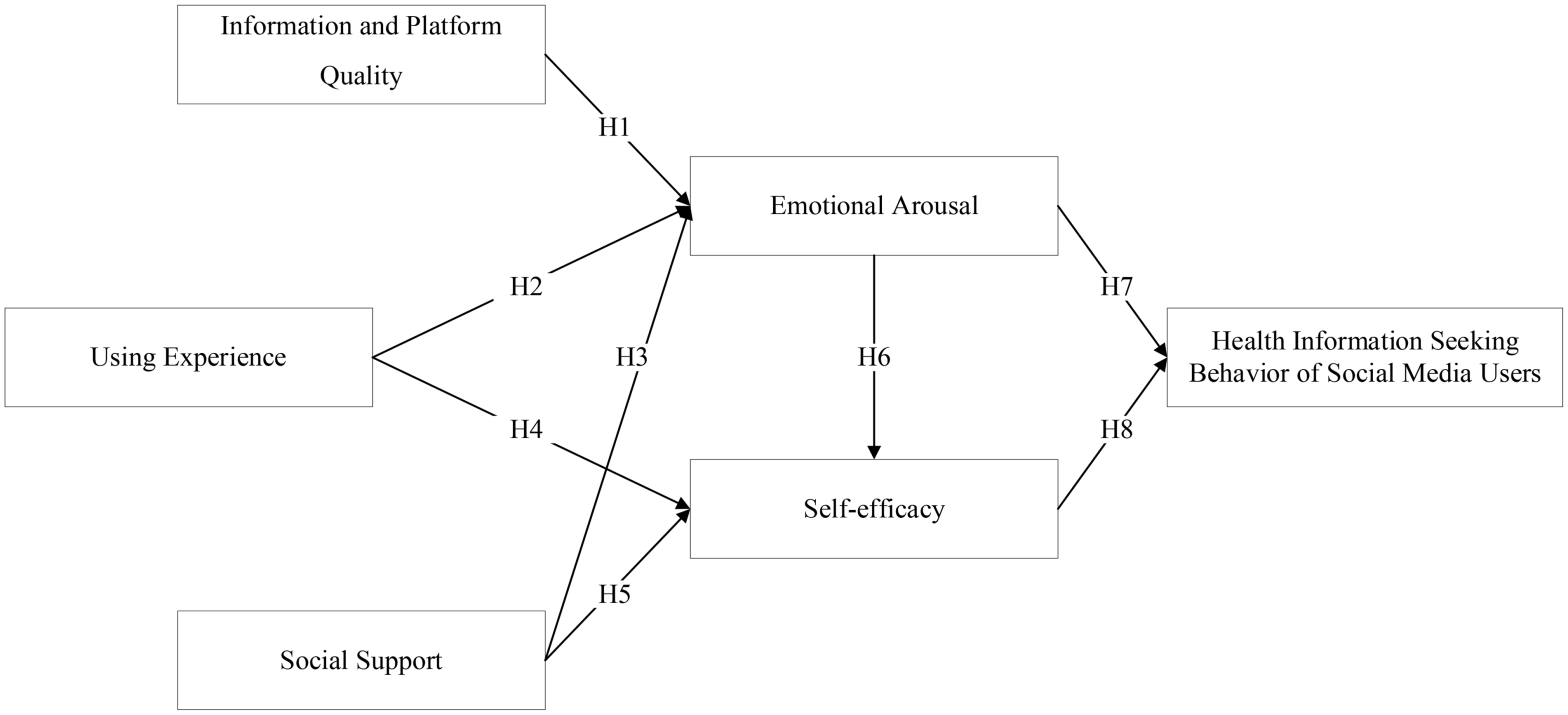
Model of influencing factors of health information seeking behavior of social media users based on social cognition theory.

Specifically, this study takes information and platform quality and social support as environmental factor variables, use experience as individual factor variables, emotional arousal and self-efficacy as cognitive process variables, and social media users’ health information seeking behavior as behavior variables, and establish a theoretical model as shown in Figure 2.

### Research hypothesis based on emotional arousal theory

Emotion is a collective term for a series of subjective cognitive feelings, which are short-lived and strong situational emotional responses (Bryant and Cox, 2006). Emotions affect people’s psychological state, thinking, and actions, and drive individuals to respond to situational stimuli, and emotions have positive or negative attributes (Chen and Ayoko, 2012). Emotional arousal refers to the physiological arousal state triggered by emotions, which is usually divided into two dimensions, valence (from pleasant to unpleasant) and activation (from excited to calm; Hadinejad et al., 2022). One of the important research methods from the perspective of emotion dimension theory is to distinguish positive emotion from negative emotion through emotional arousal, and to further analyze the relationship between emotion and behavior.

#### Cognitive processes and emotional arousal

Cognitive process refers to the process in which people acquire knowledge, process information and apply knowledge to reflect the nature of objective objects and the relationship between objects, including the forms of feeling, perception, memory, thinking, imagination, and language (Duong et al., 2021). Schachter and Singer (1962) proved through experiments that cognitive processes are dominant in the process of human emotion generation, that is, under environmental stimuli, individuals generate final emotions by explaining their own physiological emotional state changes. According to the viewpoint of social psychology, the cognitive process of an individual is mainly affected by cognitive subject factors, cognitive object factors and cognitive situational factors (Howard, 2000). Therefore, in this paper, the cognitive subject factors are reflected by the user’s experience of using social media and the experience of using social media to seeking for health information; the cognitive object factors are reflected by the quality of social media platforms and the quality of health information on the platform; the cognitive situational factors is reflected by social support. Using the three together to reflect the impact of cognitive processes on emotional arousal. Since social support not only reflects cognitive situational factors, but also reflects the influence of social ethos and behaviors of others on social media users, it will be explained in detail in section Social support and emotional arousal, and will not be repeated here. In general, the richer the user’s social media experience and health information seeking experience, the better the quality of the social media platform and the health information on the platform (Kivits, 2009; Niu et al., 2021; Jiang, 2022), and the stronger the individual user’s emotional arousal will be. Based on this, this paper proposes the following assumptions:

*H1*: Information and platform quality have a significant positive impact on emotional arousal.

*H2*: Use experience has a significant positive effect on emotional arousal.

#### Social support and emotional arousal

In addition to individual subjective cognitive process, external objective conditions are also important factors affecting emotional arousal. Jeon and Kim (2012) evaluated the relationship between service escape, emotional state and behavioral intention in international airports. The results showed that functional factors, esthetic factors, safety factors and social factors affected customers’ positive emotions, while environmental factors and social factors affected customers’ negative emotions. At the same time, the role of external objective conditions is not only reflected in the simple situational factors. From the perspective of information ecology, it not only includes the situational factors, but also contains social environmental factors and other group factors, that is, the behavior of others, social climate and wind direction are important components of external objective conditions. Lee et al. (2021) found through a survey of nursing graduate students that peer support can help graduate students overcome pressure, and peer support is positively correlated with positive achievement emotion, and negatively correlated with negative emotion. Xie and Guo (2022) explored the relationship between teacher and peer support and positive academic emotions among Chinese non-English majors in English learning. The results showed that teacher and peer support positively predicted positive academic emotions. Therefore, this paper takes “social support” as latent variable, and takes “social ethos,” “other behavior,” “situational factors” as observation variables to reflect the impact of environmental stimuli on emotional arousal. To a certain extent, a positive social ethos, repeated encouragement and persuasion and a strong desire to share, and urgent situational factors can all improve an individual’s emotional arousal. Based on this, this paper proposes the following assumptions:

*H3*: Social support has a significant positive effect on emotional arousal.

### Research hypothesis based on Bandura’s self-efficacy theory

Self-efficacy refers to an individual’s belief or skill level in coping with challenges or accepting new things (Albion et al., 2005). Under normal circumstances, people with high self-efficacy think they have a high ability to handle tasks and are confident that they can successfully complete their goals and tasks. According to Bandura (1977) self-efficacy theory, the success or failure experience of one’s own behavior, alternative experience, verbal persuasion and emotional arousal are important influencing factors of individual self-efficacy.

#### Use experience and self-efficacy

Individual direct experience is the most effective way to enhance self-efficacy (Bandura, 1977). Among these individual factors, tacit knowledge, skills and experience accumulated according to entrepreneurship, functions and industries as a result of past behaviors are the driving force to inculcate and strengthen self-efficacy (Yeo and Neal, 2006). In general, people with successful entrepreneurial experience have a higher sense of self-efficacy. Similarly, when entrepreneurs with failed entrepreneurial experience attribute failure to internal factors, it helps to emphasize cognition as the guidance of action, so that entrepreneurs can also recover confidence from failure (Benight and Bandura, 2004).

*H4*: Successful use experience has a significant positive effect on self-efficacy.

#### Social support and self-efficacy

Substitute experience refers to the experience that an individual acquires from the success or failure of the actions of others (Ali et al., 2020). Taking the health information seeking behavior of social media users as an example, when an individual sees a person of the same level as himself successfully seeking for health information through social media, he believes that he can also succeed in a similar situation, thereby improving self-efficacy (Mellor et al., 2006). Verbal persuasion refers to the support and encouragement of others to individuals, that is, others make individuals believe that they can seek for health information through social media and can successfully complete the information seeking task through reasoning, thereby improving self-efficacy. Social support usually includes support from family members, teachers and peers (Schröder et al., 2011). A study of 700 Kosovo youth by Jemini-Gashi et al. (2021) showed that social support affects career self-efficacy and career decision status. Maddy III et al. (2015) found that there is a moderate positive correlation between social support and self-efficacy, and supportive relationship can enhance self-cognition and improve re-employment prospects. As mentioned in section Model construction based on social cognitive theory, this paper uses “social support” as a latent variable to reflect the influence of others’ success or failure experience and verbal persuasion. In general, other people’s successful social media health information seeking experience and persuasion and encouragement will enhance users’ confidence in seeking for health information through social media, that is, self-efficacy will be improved. Therefore, this paper proposes the following assumptions:

*H5*: Social support has a significant positive effect on self-efficacy.

#### Emotional arousal and self-efficacy

The famous psychologist Bandura (1977) found that emotional arousal was also an important factor affecting self-efficacy in the study of “desensitization.” In the study on the relationship between positive emotional experience, negative emotional experience and self-efficacy, Medrano et al. (2016) took positive and negative emotions as a single variable and emotion regulation strategy as a covariate, and the results showed that positive and negative emotional experience would lead to the increase or decrease of self-efficacy, respectively. He and Wong (2022) investigated the influence of emotional state on creative self-efficacy and demonstrated the important role of two dimensions of emotional state (namely arousal and valence) in predicting creative self-efficacy through experimental research. Similarly, in information seeking behavior, users with high arousal positive emotions have stronger self-efficacy and action power, and they will more actively seek relevant information through social media in the face of different situations. Based on this, this paper proposes the following assumptions:

*H6*: Emotional arousal has a significant positive effect on self-efficacy.

### Research hypothesis based on ternary interactive determinism

The triadic interaction determinism takes individual, environment and behavior into consideration comprehensively, and constructs a bridge of interaction between individual’s internal cognition and external environment around behavior. It can be seen from section Research hypothesis based on emotional arousal theory and Research hypothesis based on Bandura’s self-efficacy theory that emotional arousal and self-efficacy are both affected by individual factors and environmental factors, and their state is the result of the continuous interaction between the individual, individual behavior and the environment in which the behavior occurs. Emotional arousal is the feeling of emotional intensity, such as vitality or energy, which reflects people’s judgment of the urgency and importance of information (Jennings et al., 2015). Berger and Milkman (2012) believed that emotions at a high arousal level can also contribute to a wider range of information flow, and emotional arousal has a significant role in promoting information behavior. Self-efficacy refers to the possibility that an individual perceives to be able to perform a certain health behavior and lead to a desired outcome, especially when the performed behavior requires a certain level of skill or theoretical understanding, self-efficacy becomes increasingly important (Dodel and Mesch, 2017). In the triadic interaction model of social cognitive theory, self-efficacy is one of the individual variables, which is the most important variable in this type. People tend to avoid engaging in activities that they are not confident they can perform. The more self-efficacy an individual has with Internet use, the more return he will get from online health information searching and the more active he will be in online health information seeking (Cao et al., 2016). Therefore, this paper uses emotional arousal and self-efficacy as cognitive process variables and puts forward the following hypotheses:

*H7*: Emotional arousal has a significant positive effect on health information seeking behavior.

*H8*: Self-efficacy has a significant positive effect on health information seeking behavior.

## Research method

### Questionnaire design and variable measurement

Questionnaire scales are commonly used measurement tools in sociology to indirectly measure some variables that cannot be directly measured. Based on research hypotheses and theoretical models, this research uses questionnaires to collect data. The questionnaire mainly includes three parts: questionnaire overview and concept introduction, basic information survey of social media users, and survey of social media users’ health information seeking behavior characteristics and influencing factors. Among them, the questionnaire scale uses a 5-point Likert scale to estimate each latent variable using multiple indicators. Most of the measurement indicators of the questionnaire come from the existing relevant literature. On this basis, according to the characteristics of this study, we revised the relevant indicators to improve the validity of the scale. The final variable definition and measurement items are shown in Table 1.

**Table 1:** Measurement scale of influencing factors of health information seeking behavior of social media users.

Variable	Measurement standard	Measurement item	Item source
Health information seeking behavior	Seeking channel	HISB1 I’m more likely to seeking for health information through social media than to go to the hospital to consult a doctor	Hausmann et al. (2017)
		HISB2 When I have a health problem, I actively seeking for health information through social media	
	Frequency of seeking behavior	HISB3 How often you use social media to seeking for health information	Hausmann et al. (2017)
	Network health information concern	HISB4 I will continue to follow health topics of interest and related information on social media	Cao et al. (2016)
	Seeking behavior persistence willingness	HISB5 In the future, I will maintain and even increase the frequency of seeking for health information in social media	Hausmann et al. (2017)
		HISB6 I would recommend using social media to find health information to others	
	seeking behavior identity	HISB7 Coworkers, friends, family around me think I spent a long time learning about health through social media	Mano (2014)
Information quality	Information usefulness	IQ1 The health information I found on social media was helpful	Hausmann et al. (2017)
	Information authority	IQ2 The health information I find on social media comes from authoritative sources, such as health information is a statement made by some doctors or experts	Wang and Strong (1996)
	Information relevance	IQ3 Most of the health information I find on social media is related to my “seeking terms”	Wang and Strong (1996)
	Information integrity	IQ4 The information I am seeking on social media is complete, such as both the introduction of the disease and the treatment measures	Mano (2014)
	Information legibility	IQ5 There are various types of health information in social media, including pictures, videos and other forms	Li and Wang (2018)
Platform quality	Platform capability	PQ1 Health information in social media platforms is rich in content and meets my normal needs	Li and Wang (2018)
	Personalized service	PQ2 Social media platforms can provide personalized information seeking services, such as pushing health information published by local hospitals, etc.	Li and Wang (2018)
	Privacy security	PQ3 The social media platform used does not reveal my personal information	Cao et al. (2016)
		PQ4 Browsing records on social media platforms are not collected, tracked and analyzed	
	Platform ease of operation	PQ5 Seeking for health information through social media is easy for me	Cao et al. (2016)
Using experience	Social media use experience	Exp1 Average daily time you use social media	Lin and Chang (2018)
	Social media health information seeking experience	Exp2 I am used to using social media to seeking for health information	Lin and Chang (2018)
	Success or failure of personal health information seeking	Exp3 I use social media well to find the health information I need	Parida et al. (2016)
	Health knowledge reserve	Exp4 I understand common medical knowledge and do not need to passively seeking for health information	
Social support	Social ethos	SS1 Many colleagues, family and friends around me seeking for health information through social media	Wang and Strong (1996)
	Others encourage persuasion	SS2 Many colleagues, family and friends around me support my seeking for health information through social media	Lin and Chang (2018)
	Sharing experience with others	SS3 I will select social media platforms recommended by colleagues, family members, friends or professionals (doctors, etc.) to seeking for health information	Li and Wang (2018)
	Health situational factors	SS4 When family members are unwell or there is a public health emergency, I use social media to seeking for health information	Li and Wang (2018)
	Others help	SS5 Can someone help me find answers when I am confused in health care	Kim et al. (2013)
Emotional arousal	Emotional intelligence	EA1 I am able to control my own emotions very well	Akareem et al. (2021)
		EA2 I can always see the emotions of people around me from their behavior	
		EA3 Emotions of myself or others have no influence on my ability to deal with problems rationally	
	Emotional valence	EA4 I am in a good mood about the experience of seeking for health information through social media	Akareem et al. (2021)
	Perceived satisfaction	EA5 I am satisfied with getting the health information I need through social media	Kim et al. (2013)
	Emotional activation	EA6 Using social media to find health information makes me nervous	Akareem et al. (2021)
Self-efficacy	Perceived confidence	Se1 I am confident that I can find health information through social media and use it effectively	Bandura (1977)
	Perceived advantage	Se2 When seeking for health information through social media, I always find relevant information before others	
	Friends appreciate	Se3 Colleagues, friends, and family around me think I’m good at finding health information through social media	
	Seeking persistence	Se4 When my seeking for health information through social media fails, I keep seeking instead of giving up	

### Data collection and statistical methods

The research object of this paper is the user groups of social media platforms, including comprehensive question-and-answer platforms (such as Baidu Zhizhi, Zhihu, Sogou Ask), online consulting platforms (such as WeDoctor, Chunyu Doctor, and Haoda Doctor Online) and professional health information website forums.

To ensure the reliability and validity of the research content, a two-stage questionnaire was used in this study. In the first stage, we consulted the existing relevant literature and combined with the research focus of this paper to form the first draft of the questionnaire. Then three experts in the field of health information behavior were invited to give feedback on the questionnaire to form a pre-survey questionnaire. We then conducted a small sample survey and exploratory factor analysis to improve the initial design of the questionnaire. In the second stage, we used the revised questionnaire to conduct a formal questionnaire survey. The revisions are mainly reflected in the change of “monthly average income” to “monthly average expenditure” and the change of “content communities such as Youku and Guokke” to “content communities such as Douyin and Bilibili.” Exchange the order in which “Do you have social media health information search experience” and “the type of health information you care about” are presented. On this basis, confirmatory factor analysis was used to delete relevant questionnaire items to form the final scale.

We administered the formal questionnaire in two ways. One is to design online questionnaires through the Questionnaire Star platform, and distribute questionnaires to social media users through QQ, WeChat, Zhihu, Baidu Post Bar, etc. The second is to design a questionnaire identical to the questionnaire star through Credamo platform and conduct the survey through Credamo questionnaire community. A total of 252 questionnaires were returned in this study. We eliminated the questionnaires with high answer consistency through variance screening, and 219 valid questionnaires were obtained. The effective rate of the questionnaire was 86.9%. Of these, 181 samples had social media search experience for health information and 28 samples had no social media search experience. Finally, the empirical study verifies the model and hypothesis established in this paper.

## Empirical analysis and research results

### Descriptive statistical analysis

The paper uses SPSS 28.0 to perform descriptive statistical analysis on the personal basic situation of social media users in the sample (see Table 2). Among them, the ratio of males to females is about 4:6, and the research samples are mainly users who are 18–40 years old, have a bachelor’s degree or above, and have an average monthly expenditure of 1,000–5,000 yuan. By comparing the use of social media to obtain health information and did not use social media to obtain information of the basic information of the respondents, we can find that using social media time in a day on average 1.5 h, the experience for such information of the users, users with college or above major in cultural level is relatively high. In the samples without such experience, the majority of users with education level are high school and below, which indicates that users with rich experience in using social media or with higher education are more inclined to use social media to seeking for health information. Since most users with high education or rich experience in using social media have higher health information literacy and information retrieval ability, this result reflects the frequency of users with higher health information literacy and information retrieval ability who use social media to seeking for health information higher. In addition, the main reason given by users who did not use social media to seeking for health information was “not often getting sick,” that is, lack of a direct motivation to seeking for health information is an important reason why some users refuse to use social media to seeking for health information.

**Table 2:** Basic personal information of sample social media users.

Basic personal information	Category	Total sample (*n* = 219)		Samples who have used social media to seeking for health information (*n* = 181)	
		Quantity	Frequency (%)	Quantity	Frequency (%)
Gender	Male	85	38.8	73	40.3
	Female	134	61.2	108	59.7
Age	18~25 years old	70	32.0	67	37.0
	26~30 years old	30	13.7	29	16.0
	31~40 years old	46	21.0	36	19.9
	41~50 years old	59	26.9	40	22.1
	Over 50 years old	14	6.4	9	5.0
Education	Junior high school and below	24	11.0	8	4.4
	High school, Technical school, Secondary school	41	18.7	28	15.5
	College	19	8.7	17	9.4
	Undergraduate	112	51.1	105	58.0
	Graduate and above	23	10.5	23	12.7
Average monthly expenditure	0~1,000 RMB	22	10.0	16	8.8
	1,001~3,000 RMB	95	43.4	84	46.4
	3,001~5,000 RMB	53	24.2	42	23.2
	5,001~8,000 RMB	34	15.5	29	16.0
	More than 8,000 RMB	15	6.8	10	5.5

In terms of seeking preference, the respondents paid more attention to healthy life information (90.4%), mental health information (56.2%) and disease diagnosis information (70.3%). It may be that chronic diseases are becoming more common and difficult to cure due to people’s increasing work pressure and irregular life. At the same time, due to the impact of the new crown epidemic, people’s medical and health awareness has increased, and people pay more attention to health care issues in daily life. They want to easily meet their health information needs through social media. At the same time, due to the popularization of mental health knowledge in recent years, such health information has gradually become one of the focuses of users’ attention.

### Measurement model checking

This paper mainly uses SPSS 28.0 and Amos 24.0 to evaluate the rationality of the measurement model and the structural model, and to test the theoretical hypothesis proposed in this study. Since the main purpose of this study is to verify the model hypothesis, the covariance structural equation model that fits the off-diagonal elements of the observed variable covariance matrix better is selected. Compared with the structural equation model based on partial least squares, its parameter estimation is more accurate. The reliability and validity of the questionnaire can analyze the measurement quality of the questionnaire. The test results are shown in Table 3.

**Table 3:** Reliability and validity tests.

Latent variable	Information and platform quality	Using experience	Social support	Emotional arousal	Self-efficacy	Health information seeking behavior
Information and platform quality	**0.690**					
Using experience	0.000	**0.698**				
Social support	0.000	0.000	**0.701**			
Emotional arousal	0.522	0.509	0.000	**0.712**		
Self-efficacy	0.184	0.696	0.173	0.615	**0.763**	
Health information seeking behavior	0.254	0.533	0.095	0.632	0.731	**0.789**

Note: The statistics in bold type are the square root values of AVE, and the data below them are the Pearson correlations of the dimension.

From the reliability test of the questionnaire, all the Cronbach’s alpha coefficients in the measurement model are greater than the recommended value of 0.7, indicating that the questionnaire has good stability and internal consistency, and can reliably measure latent variables. Validity test measures whether the project design is reasonable, and it is divided into content validity and construct validity. First, in terms of content validity, most of the measurement items in this paper are adapted from the existing literature and get expert feedback before the formal investigation, so the content validity is good. Second, construct validity is an important indicator, which can be divided into convergent validity and discriminant validity. As for convergent validity, the factor loadings of each observed variable are all around 0.6 (see Table 4), indicating that the model has a high correlation between the observed variables and the structural variables to which they belong. The CR values of the model all exceeded the recommended value of 0.7, showing that the internal consistency of the model variables was good, and the set observation variables could better explain the underlying structural model. In addition, the AVE values in this model are all around 0.5, revealing that the observed variables can better explain each measurement dimension. From the perspective of discriminant validity, the square root of the AVE value of each observation variable is greater than the correlation coefficient between it and other observation variables, indicating that each observation variable has a strong discriminant coefficient and a high degree of discrimination.

**Table 4:** Latent variable factor loadings.

Latent variable	Variable item	Factor loadings	Cronbach’s alpha	CR	AVE
Information and platform quality	IQ&PQ 1	0.814	0.705	0.722	0.476
	IQ&PQ 2	0.735			
	IQ&PQ 3	0.474			
Using experience	Exp 1	0.656	0.710	0.740	0.488
	Exp 2	0.760			
	Exp 3	0.675			
Social support	SS 1	0.683	0.826	0.829	0.492
	SS 2	0.678			
	SS 3	0.695			
	SS 4	0.692			
	SS 5	0.756			
Emotional arousal	EA 1	0.823	0.792	0.802	0.507
	EA 2	0.698			
	EA 3	0.588			
	EA 4	0.720			
Self-efficacy	Se 1	0.766	0.792	0.735	0.581
	Se 2	0.759			
Health information seeking behavior	HISB 1	0.688	0.779	0.765	0.623
	HISB 2	0.879			

### Structural model analysis

**Figure 3:**
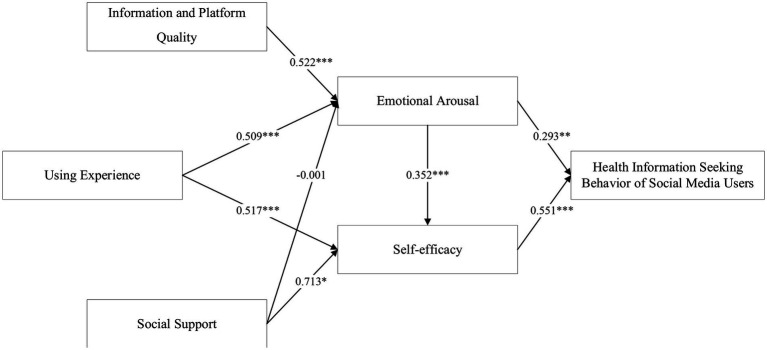
Model fitting results. ^*^means *p* < 0.05, ^**^means *p* < 0.01, ^***^means *p* < 0.001.

Structural models can not only measure the relationship between indicators and latent variables, but also measure the causal relationship between variables in the model. This study uses Amos 24.0 software as a structural equation model analysis tool to test the significance of the path coefficients of the observed variables on the health information seeking behavior of social media users. The analysis results are shown in Figure 3 and Table 5.

**Table 5:** Summary of hypothesis testing results.

Hypothesis	Inspection result
H1: Information and platform quality have a significant positive impact on emotional arousal.	Supported
H2: Use experience has a significant positive effect on emotional arousal.	Supported
H3: Social support has a significant positive effect on emotional arousal.	Not supported
H4: Successful use experience has a significant positive effect on self-efficacy.	Supported
H5: Social support has a significant positive effect on self-efficacy.	Supported
H6: Emotional arousal has a significant positive effect on self-efficacy.	Supported
H7: Emotional arousal has a significant positive effect on health information seeking behavior.	Supported
H8: Self-efficacy has a significant positive effect on health information seeking behavior.	Supported

Based on the above model results, we can draw the following conclusions. In terms of the path coefficients and their significance levels, the relationships proposed in this paper are significant except for the H3 (that social support has a significant positive effect on emotional arousal), which is not supported. H1, H2, H4, H6, and H8 all reach the significance level of 0.001, and the path coefficients are 0.522, 0.509, 0.517, 0.352, and 0.551, respectively. H7 reaches the significance level of 0.01, and the path coefficient is 0.293. H5 reaches the significance level of 0.05, and the path coefficient is 0.713. The results of the study show that the influence of self-efficacy on users’ information seeking behavior is significantly higher than that of emotional arousal. The main reason is that emotional arousal emphasizes the physiological arousal state triggered by emotion, rather than the direct driving effect on behavior. Although positive emotional arousal can make users have a unique emotional experience and can directly further stimulate the generation of seeking behaviors, its main role is to promote users to maintain a state of passion and confidence, that is, to improve user self-efficacy. Furthermore, the hypothesis from social support (H3) to emotional arousal was not tested. Possibly due to the weak group nature of social media user groups, the social atmosphere of the platform and the behavior of netizens or people around them have little impact on the emotional attitudes of social media users about their health information seeking behavior, so this phenomenon needs further research.

## Conclusion

Under the dual background of “Internet + medical and healthcare” and the trend of standardized COVID-19 management, this study explores the influencing factors of social media users’ health information seeking based on social cognition theory. In this study, we constructed a theoretical model and proposed research hypotheses by taking information and platform quality and social support as environmental factors, using experience as individual factors, emotional arousal and self-efficacy as cognitive process variables, and health information seeking behavior of social media users as behavioral variables. The research collects empirical data through the questionnaire method, and uses the covariance structural equation model to test. The results show that the model fits the data well. From the path coefficient and its significance level, except for H3 (social support has a significant positive impact on emotional arousal), all other relationships proposed in this paper are significant, and the following conclusions can be drawn:

1. The quality of social media platforms, the quality of health information on the platforms, and the richness of individual use experience are all important factors that affect the emotions of social media users, which are consistent with the conclusions of existing studies (Schachter and Singer, 1962; Kivits, 2009; Niu et al., 2021; Jiang, 2022). At the same time, it broadens the field of health information research and projects the perspective to the context of the epidemic. It shows that the better the quality of social media platforms and the quality of health information on the platform, and the more experience users have in using social media to successfully seeking for the health information they need, the stronger the emotions that users are willing to use social media to seeking for health information.
2. The richness of individual experience, the level of social support, and the user’s emotions about using social media to seeking for health information are all important factors that affect self-efficacy, which is consistent with previous research results (Schröder et al., 2011; He and Wong, 2022). It shows that the more successful experience of using social media to seeking for health information, the higher the level of social support they can receive, and the more positive and strong emotions they are willing to use social media to seeking for health information, the stronger their self-efficacy will be.
3. Users’ emotions and self-efficacy toward using social media to seeking for health information are important factors that affect the health information seeking behavior of social media users. These are the two core conclusions of this paper, which clarify the important factors that affect the health information seeking behavior of social media users from the root. It shows that under the background of the current huge variety of information sources and diversified information acquisition methods, users with strong emotions about using social media to seeking for health information are more likely to use this method to obtain the health information they need. Users with strong self-efficacy are more confident in their ability to use social media and obtain health information, and are more likely to seeking for health information through social media.

In theory, based on triadic interaction determinism and combining with the view of information ecology, this paper focuses on the positive influence path from environmental and individual factors to cognitive processes to behaviors. This paper summarizes the theory-driven research model and verifies the effectiveness of the model through empirical data, revealing the relationship between social media users’ health information seeking behavior and personal and environmental factors under the pandemic. This study lays the foundation for the future related research. From the perspective of research, compared with the existing research on health information seeking behavior, this paper broadens the research perspective of information seeking behavior and enriches the research on information seeking behavior in social cognitive theory. From the perspective of search actors, the content and characteristics of public health information search behavior were seriously affected by the COVID-19 epidemic. This paper can provide reference for the research of online health information seeking behavior of different groups. From the perspective of influencing factors, future related research can proceed from the theme or specific influencing factors in the model constructed in this paper to conduct in-depth research on the influencing factors of online health information seeking behavior of social media users.

In practice, according to the conclusion of this study, we can make suggestions from three aspects. At the user level, social media users should constantly accumulate their own successful experience in seeking health information, actively learn from friends and relatives, humbly accept help from others, and maintain positive and optimistic emotions, which will continuously improve their self-efficacy and generate more frequent health information searching behavior on social media. At the platform level, platform operators should further improve platform performance and strengthen health information monitoring. As an online health information provider, the platform should strictly control the quality of health information, improve the comprehensiveness and integrity of health information, and develop customized personalized health information services for different demanders to improve the experience satisfaction of users. At the government level, the government should actively promote the integration of health and the Internet, develop online medical services to help alleviate the imbalance of medical resources supply, and standardize the management of health service platforms. At the same time, the government should also cultivate a healthy and civilized social atmosphere and create a good ecological environment for health information seeking. However, it is worth noting that when the model of this study is extended and applied to other groups, the differences in the characteristics of the actors and the searching environment should be considered, and then the model should be adjusted and modified.

In addition, there are still some limitations of this study that need to be further addressed in future studies. First, due to factors such as the way of questionnaire distribution, the educational background of the research sample is generally higher than that of current social media users. Users with different educational backgrounds have different abilities to seeking for health information using social media, different emotional control abilities, and different self-efficacy, which will have a certain impact on the research results. Secondly, the questionnaire survey method is greatly influenced by the user’s subjectivity, which leads to certain errors in the measurement. Therefore, in future research, we will refine the research questions and consider different types of user groups to make the research results more universal and scientific. In addition, we can comprehensively use the temporal information behavior analysis method to deepen the research on this topic. Finally, we will consider a combination of structured interviews, text analysis and other methods for optimization to obtain more reliable research results.

## Data availability statement

The raw data supporting the conclusions of this article will be made available by the authors, without undue reservation.

## Ethics statement

Ethical review and approval was not required for the study on human participants in accordance with the local legislation and institutional requirements. The patients/participants provided their written informed consent to participate in this study. Written informed consent was obtained from the individual(s) for the publication of any potentially identifiable images or data included in this article.

## Author contributions

XZ: designing. BC and GL: writing. YD: method. All authors contributed to the article and approved the submitted version.

## Funding

This work was supported by National Social Science Foundation of China (21BTQ059) (Research on Information Behavior Preference Feature Mining and Recommendation Based on Users’ Cross-Social Media).

## Conflict of interest

The authors declare that the research was conducted in the absence of any commercial or financial relationships that could be construed as a potential conflict of interest.

## Publisher’s note

All claims expressed in this article are solely those of the authors and do not necessarily represent those of their affiliated organizations, or those of the publisher, the editors and the reviewers. Any product that may be evaluated in this article, or claim that may be made by its manufacturer, is not guaranteed or endorsed by the publisher.
